# Cognitive–psychological mechanisms and decision-making in later life: the role of wealth perception capability

**DOI:** 10.3389/fpsyg.2026.1843925

**Published:** 2026-05-20

**Authors:** Xin He, Xin Zeng, Tao Jiang

**Affiliations:** 1 School of Finance, Southwestern University of Finance and Economics, Chengdu, China; 2 Research Institute of Economics and Management, Southwestern University of Finance and Economics, Chengdu, China; 3 Survey and Research Center for China Household Finance, Southwestern University of Finance and Economics, Chengdu, China

**Keywords:** consumer psychology, consumption decision-making, older adults, perceived wealth, psycho-cognitive mechanisms

## Abstract

**Introduction:**

Decision-making in later life is shaped not only by physiological decline but also by the joint influence of cognitive and psychological factors. This study focuses on wealth perception capability and examines how older adults’ subjective perceptions of their own financial circumstances affect their engagement in decision-making. By moving beyond the conventional emphasis on physical aging and functional decline, this study seeks to extend our understanding of the cognitive–psychological mechanisms underlying decision-making in later life.

**Methods:**

Using micro-level survey data covering tens of thousands of elderly households, together with individuals’ self-reported psychological information, this study constructs a household-level measure of wealth perception capability. Compared with other types of decision-making, consumption behavior is more susceptible to cognitive and emotional influences; therefore, this study selects consumption decisions as the core domain for examining decision-making among older adults. On this basis, two-way fixed-effects models and two-stage least squares estimation are employed to test the effect of wealth perception capability on older adults’ decision engagement and its underlying mechanisms. Heterogeneity across different groups of older adults is also examined.

**Results:**

The results show that improvements in wealth perception capability significantly enhance decision engagement among elderly households at the 1% statistical level. Mechanism analysis indicates that wealth perception capability operates primarily by reshaping individuals’ subjective perceptions of their financial situation. Specifically, higher wealth perception capability strengthens perceived financial control and financial security, alleviates psychological constraints, and reduces excessive precautionary motives driven by uncertainty, thereby encouraging older adults to participate more actively in consumption-related decisions. Heterogeneity analysis further reveals that this effect is more pronounced among rural households, older adults living alone or lacking family support, and those with lower levels of cognitive health. These findings suggest that wealth perception capability plays a stronger compensatory role among psychologically vulnerable groups.

**Discussion:**

These findings underscore the importance of subjective psychological perception in shaping decision-making behavior in later life. In contrast to prior research that has primarily focused on the effects of physiological decline on older adults’ decision-making capacity, this study shows that, in the context of digital development, wealth perception capability represents an important cognitive–psychological factor influencing decision engagement among older adults. By revealing the psychological mechanisms through which wealth perception capability affects consumption decisions, this study deepens our understanding of decision-making in later life and provides useful implications for promoting cognitive well-being, financial confidence, and mental health among older adults.

## 1 Introduction

Consumption decision-making is among the types of decisions most susceptible to cognitive and emotional influences in older adults. From a cognitive processing perspective, individuals must integrate multiple sources of information when making consumption choices, including current resource constraints, future uncertainty, and subjective expectations. This process relies heavily on subjective evaluations of risk and return. Compared with younger individuals, older adults exhibit systematic differences in cognitive functioning, information processing capacity, and risk perception, making their decision-making processes more vulnerable to emotional states and subjective judgments. In later life, individuals typically face greater longevity risk, health uncertainty, and income instability. As a result, their consumption decisions depend more strongly on psychological evaluation mechanisms than solely on objective wealth levels. In this context, psychological factors such as financial security, precautionary motives, and perceived control play a critical role. Among these, perceived wealth—defined as individuals’ subjective evaluation of their financial situation—serves as a fundamental determinant of consumption behavior under uncertainty. Even when objective wealth levels are identical, differences in subjective perceptions can lead to substantial variation in consumption tendencies. This suggests that consumption behavior reflects not only resource constraints but also underlying psychological evaluation processes.

In the context of a digital society, the process of wealth perception is undergoing profound changes. In this study, wealth perception capability is defined as the cognitive ability to perceive, interpret, and evaluate one’s own wealth, which fundamentally relies on information acquisition, information processing, and cognitive integration. With the deep integration of digital technologies into daily life, internet-enabled devices have reduced the cost of information acquisition and enhanced the visibility and accessibility of personal assets, thereby reshaping how older adults perceive their wealth (Eshet, 2004; Hsieh et al., 2008; Brandtzæg et al., 2011). For instance, the proliferation of digital payment records, online financial accounts, and smart devices allows individuals to access wealth-related information more frequently and intuitively, thereby strengthening wealth perception. This process fundamentally alters individuals’ subjective assessment of resource availability.

However, substantial heterogeneity in wealth perception capability exists across individuals and exhibits clear structural patterns. Prior research shows that education (Zhong, 2011), income (Cruz-Jesus et al., 2018), gender and age structure (Mumporeze and Prieler, 2017), residential location (Shelton et al., 2015), and infrastructure conditions (Büchi et al., 2016) significantly influence individuals’ ability to access and use information technologies, thereby shaping disparities in wealth perception capability. Low-income groups and individuals facing higher costs of technology adoption are structurally disadvantaged in the digital society (Ihm and Hsieh, 2015). As a vulnerable group, older adults face greater barriers to accessing and using digital technologies due to physiological decline and deteriorating health, which constrains their wealth perception capability (Niehaves and Plattfaut, 2014). Moreover, insufficient consideration of cognitive decline and reduced information-processing capacity in current digital product design further exacerbates their disadvantage in the wealth perception process. These constraints not only limit older adults’ objective participation in digital economic activities but also reinforce behavioral constraints through psychological mechanisms. Specifically, lower perceived control may weaken individuals’ confidence in the future, while heightened perceptions of uncertainty may strengthen precautionary saving motives, thereby suppressing consumption. This indicates that wealth perception capability affects not only how individuals understand their resources but also how psychological constraints—such as perceived control and uncertainty expectations—shape their decision-making.

Against the backdrop of deepening population aging and rapid digital technological development, enhancing wealth perception capability may represent a critical pathway to improving decision-making among older adults. However, the existing literature has paid relatively limited attention to micro-level psychological mechanisms, particularly in the context of elderly decision-making. An important yet underexplored question, therefore, is whether wealth perception capability influences consumption decision-making among older adults through psychological pathways. To address this gap, this study uses data from the 2017, 2019, and 2021 China Household Finance Survey (CHFS) to construct a comprehensive household-level index of wealth perception capability and systematically examine its impact on consumption decisions among elderly households.

This study makes two main contributions. First, existing research has placed greater emphasis on the effects of objective environmental constraints and physiological decline on older adults (Feng et al., 2025), whereas decision-making in later life is often shaped jointly by emotional states, cognitive aging, and subjective cognitive evaluation (Mikels and Taullahu, 2023). By comparison, relatively limited attention has been paid to the role of psychological and cognitive processes in older adults’ economic decision-making. From a psychological and cognitive perspective, the present study centers on wealth perception capability and highlights the critical role of subjective perception in shaping decision-making among older adults. Second, existing studies on older adults’ perceptual capabilities have tended to rely on single-dimensional measures. Building on prior research (Choi et al., 2023), this study develops a multidimensional measure of household wealth perception capability, including access breadth, access depth, usage breadth, and usage depth, thereby providing a more comprehensive framework for capturing older adults’ cognitive capacity in digital environments.

## 2 Theory and hypotheses

The effect of wealth perception capability on consumption among elderly households can be understood as a cognitively based psychological–behavioral process. According to life-cycle theory, individuals are expected to allocate consumption and savings intertemporally across different stages of life in ways that maximize lifetime utility. In later life, however, as labor income declines, accumulated household wealth becomes increasingly important for maintaining living standards and well-being. Under these conditions, consumption decisions depend not only on objective economic resources but also on individuals’ ability to perceive, interpret, and evaluate those resources accurately. Prior research suggests that, relative to objective wealth, subjective wealth perception may exert a more direct influence on consumption confidence and willingness to spend (Kivetz, 1999). At the same time, advances in science and technology, together with the growing integration of digital technologies into everyday life, may improve convenience, subjective well-being, and quality of life (Lei et al., 2023). For older adults, however, greater awareness of future living arrangements and survival prospects does not necessarily lead to higher current consumption, because consumption behavior is often shaped by subjective perceptions of resource adequacy and confidence in financial decision-making. In particular, greater risk aversion, age-related cognitive decline, and limited information-processing capacity may prevent older individuals from fully translating objective wealth into actual consumption, even when their material conditions are adequate. By improving the ability to identify, integrate, and interpret household wealth information, wealth perception capability may increase the perceived availability of disposable resources, reduce psychological frictions in decision-making, and facilitate the conversion of latent consumption demand into actual expenditure. Accordingly, this study proposes that wealth perception capability is positively associated with consumption among elderly households.

*H1: Household wealth perception capability is positively associated with elderly household consumption.*

One important psychological mechanism through which wealth perception capability may influence consumption is perceived financial control. From a micro-level decision-making perspective, household consumption depends not only on the amount of wealth a household holds, but also on whether individuals believe they can manage and use that wealth effectively in response to present and future needs. Older adults often allocate a larger share of their assets to safer but less liquid and less readily observable forms. Combined with liquidity constraints, this asset structure may weaken their subjective sense of disposable wealth and, in turn, suppress consumption. In this sense, even when objective resources are sufficient, limited confidence in one’s financial capacity may reduce the willingness to use those resources for current spending. Greater wealth perception capability allows individuals to better understand the composition, liquidity, and sustainability of household wealth, reduces information asymmetry and cognitive bias, and strengthens the sense that they can exercise control over their financial lives. As perceived financial control increases, older adults may feel more confident in their spending decisions and become more willing to translate potential purchasing power into actual expenditure. Thus, perceived financial control is likely to be an important psychological pathway linking wealth perception capability to consumption among elderly households.

*H2: The positive association between household wealth perception capability and elderly household consumption is mediated by perceived financial control.*

A second important psychological mechanism is the reduction of excessive precautionary saving motives. From the perspective of life-cycle theory, uncertainty about future income generally declines in old age, and precautionary saving motives should therefore weaken. In practice, however, this prediction may be moderated by psychological factors. Under conditions of heightened uncertainty and salient risk perception, subjective judgments may depart from the rational optimum and thereby shape consumption decisions. As noted above, the widespread integration of digital technologies into daily life may strengthen positive expectations about future living conditions and increase individuals’ subjective assessments of survival probability (Lei et al., 2023). As a result, perceptions of longevity risk may become more salient among older adults, reinforcing precautionary saving motives and encouraging households to retain more resources against possible future contingencies, thereby suppressing current consumption. More importantly, when wealth perception capability is limited, elderly households may underestimate the adequacy and accessibility of their actual resources while overestimating future financial risks, causing precautionary motives to become psychologically excessive. By contrast, stronger wealth perception capability may enable individuals to assess their financial position more accurately, reduce subjective uncertainty about the future, and weaken overly conservative saving behavior driven by misperceived insecurity. As such unnecessary psychological caution recedes, households may become more willing to release current consumption potential. Therefore, this study proposes that wealth perception capability may promote consumption among elderly households by reducing excessive precautionary saving motives.

*H3: The positive association between household wealth perception capability and elderly household consumption is mediated by the reduction of excessive precautionary saving motives.*

## 3 Data and variables

### 3.1 Data

This study draws on both micro-level elderly household data and province- and city-level macroeconomic data. The micro-level data are obtained from the 2017, 2019, and 2021 waves of the China Household Finance Survey (CHFS), conducted by the Survey and Research Center for China Household Finance at Southwestern University of Finance and Economics. The CHFS covers 29 provincial-level administrative regions, 355 counties (districts), and 1,481 communities, and provides rich information on individual characteristics—such as gender and employment status—as well as household-level indicators, including income, expenditure, assets, liabilities, and insurance coverage. Provincial-level control variables are sourced from the National Bureau of Statistics of China’s provincial annual database, while instrumental-variable data are drawn from prefecture-level city statistical yearbooks.

To ensure data quality, observations with missing values for key variables or obvious anomalies are excluded. In addition, continuous variables—including per capita household consumption expenditure, per capita net income, and per capita liabilities—are winsorized at the 0.1% level and transformed using natural logarithms. After these procedures, the final sample consists of 31,662 elderly households.

### 3.2 Variables

#### 3.2.1 Independent variables

The key explanatory variable in this study is the wealth perception capability of elderly households. To capture both the absolute and relative dimensions of this construct, we employ two complementary measures: the household wealth perception capability index and the wealth perception gap index. These indices are constructed using the full sample of households from the 2017, 2019, and 2021 waves of the China Household Finance Survey (CHFS), including both elderly and non-elderly households, in order to more accurately position elderly households within the broader digital financial context.

First, the wealth perception capability index is constructed to measure the absolute level of wealth perception capability among elderly households. Unlike objective wealth or general financial literacy, wealth perception capability emphasizes individuals’ subjective appraisal and overall understanding of the adequacy, accessibility, and constraints of their financial resources. In this sense, it reflects a capability that is simultaneously cognitive, context-dependent, and behaviorally oriented (Tully and Sharma, 2022). Precisely because of this multidimensional nature, the construct cannot be adequately captured by a single indicator, but instead requires a more systematic and comprehensive measure. To this end, the present study draws on the multidimensional framework of access, capability, and utilization proposed by Jun et al. (2025), while also incorporating insights from research on older adults’ digital access and digital engagement (Rodríguez-Miranda et al., 2025; Yang et al., 2025). We develop a measurement system for household wealth perception capability along two dimensions, namely breadth and depth. This approach makes it possible to capture more precisely the psychological capacity of older adults to perceive and interpret wealth across multiple domains of everyday life in a digital society. The detailed indicator system is reported in Table 1, and the index is constructed using the entropy-weighting method.

**Table 1:** Measurement Index System for household wealth perception capability.

Primary indicators	Secondary indicators	Tertiary indicators	Measurement question
Access capability	Access breadth		Household ownership of a mobile phone
			Computer ownership
			Smartphone ownership
	Access depth		Number of years household has had smartphone access
Usage proficiency	Usage breadth	Digital business	Whether the household operates an online business
			Whether production and business loans are applied for online
		Digital living	Whether there are online lending activities beyond business loans
			Whether the household engages in non-financial online purchases or sales
		Digital wealth management	Whether financial products are purchased online
			Ownership of a third-party payment account
	Usage depth	Digital operations	Maximum online sales revenue from a single business project
			Largest online loan amount borrowed by the household for business purposes
		Digital living	Annual household expenditure on online shopping
		Digital wealth management	Balance of household third-party payment accounts

Second, the household wealth perception gap index captures the relative dimension of wealth perception capability and is intended to measure the extent to which elderly households lag behind high-capability households within the sample (Qin et al., 2022). The disadvantage faced by elderly households in wealth perception is reflected not only in their lower level of capability, but also in their relatively disadvantaged position within the overall distribution. Focusing exclusively on the absolute level of wealth perception capability may therefore be insufficient to fully capture the relative disparities between elderly households and other groups in terms of wealth appraisal, resource accessibility, and adaptation to the cognitive demands of a digital environment. To address this limitation, and following the approach of Gero et al. (2022), we further construct a household wealth perception gap index to complement the absolute measure of wealth perception capability, thereby providing a more comprehensive account of the cognitive constraints and potential inequalities faced by elderly households. To reduce the influence of extreme values, the 1st and 99th percentiles of the household wealth perception capability index are used to replace extreme values in the construction of the wealth perception gap index. The calculation procedure is presented in Equation 1. This index captures the relative distance between a household’s wealth perception capability and the upper bound of the distribution. A higher value indicates a lower relative level of wealth perception capability.


$$\text{divid}e_{\mathrm{ij}}=\frac{\text{high}\_\mathrm{per}(\text{digita}l_{i})-\text{digita}l_{\mathrm{ij}}}{\text{high}\_\mathrm{per}(\text{digi}tal_{i})-\mathrm{low}\_\mathrm{per}(\text{digita}l_{i})}$$ (1)

In Equation 1, divide_ij_ denotes the wealth perception gap index for elderly household j in year i, denotes the wealth perception capability index for elderly household j in year i, high_per(digital_i_) denotes the 99th percentile of the household wealth perception capability index in year i, and low_per(digital_i_) denotes the 1st percentile of the household wealth perception capability index in year i.

#### 3.2.2 Dependent variables

The dependent variable in this study is elderly household consumption, measured as the natural logarithm of per capita household consumption expenditure. The CHFS provides detailed information on consumption across multiple categories, including food and tobacco, clothing, housing, household equipment and services, transportation and communication, education and entertainment, healthcare, and other expenditures. In line with the consumption characteristics of older adults, consumption is classified into three broad categories: basic consumption (food and tobacco, clothing, and housing), medical consumption (healthcare), and enjoyment-oriented consumption (education and entertainment, transportation and communication, household equipment and services, and other expenditures).

Given the relatively rigid nature of medical expenditures, the core consumption measure used in this study is per capita household consumption expenditure excluding medical consumption. As the consumption information in the CHFS refers to expenditures in the previous year, the three survey waves correspond to consumption in 2016, 2018, and 2020, respectively.

#### 3.2.3 Control variables

This study includes a set of control variables at three levels: household characteristics, household head characteristics, and regional characteristics. Household-level controls comprise annual per capita net household income, per capita household liabilities, per capita household assets, and household size. The characteristics of the household head include gender, age, health status, marital status, employment status, and years of schooling. Regional-level controls include the old-age dependency ratio, per capita GDP, and the value added of the tertiary sector in the province where the household is located. For years in which provincial-level old-age dependency ratios are unavailable, missing values are imputed using the average of adjacent years.

#### 3.2.4 Descriptive statistics

Table 2 reports the descriptive statistics for the main variables used in the regression analysis. The results indicate that the mean value of the wealth perception capability index for elderly households is below 0.1, indicating that, relative to other groups, elderly households still exhibit a lower level of wealth perception capability.

**Table 2:** Descriptive statistics of variables.

Variable name	Definition	Sample size	Mean	Standard deviation
Household Wealth Perception Capability Index	Constructed using the entropy method	31,662	0.019	0.026
Household Wealth Perception Gap Index	Calculation method detailed in variable definition	31,662	0.878	0.167
Elderly Household Consumption	Per capita household consumption, calculated as total household consumption expenditure divided by total household population (yuan)	31,662	18475.732	24234.784
Household Income	Total household income divided by total number of household members (yuan)	31,662	27383.404	33956.103
Household Assets	Total household assets divided by total number of household members (yuan)	31,662	480474.1	1174602.316
Household Debt	Total household debt divided by total household members (yuan)	31,662	5255.146	28978.897
Household Size	Total number of household members	31,662	2.62	1.522
Age	Age of head of household (years)	31,662	68.703	6.826
Health Status	Household head health status, 1 = Very unhealthy; 2 = Fairly unhealthy; 3 = Average; 4 = Fairly healthy; 5 = Very healthy	31,662	3.093	1.014
Marital Status	Whether the head of household is married. 0 = Unmarried; 1 = Married	31,662	0.976	0.152
Work Status	Whether the head of household is employed. 0 = Not employed; 1 = Employed	31,662	0.337	0.473
Gender	Household head’s gender, 0 = female; 1 = male	31,662	0.740	0.438
Education Status	Head of household’s years of education, converted based on educational attainment (years)	31,662	7.907	4.155
Provincial Elderly Dependency Ratio	Provincial old-age dependency ratio	31,662	0.164	0.032
GDP	Provincial GDP per capita (yuan)	31,662	67159.824	31454.290
Provincial Tertiary Industry Value-Added	Provincial tertiary industry value-added (billion)	31,662	17904.876	12354.904

This table presents the descriptive statistics of the explanatory variables, dependent variables, and control variables in this paper. This paper employs the logarithmic values of per capita household consumption, per capita household income, per capita household assets, per capita household debt, provincial per capita GDP, and provincial tertiary industry value-added in regression analysis.

### 3.3 Model specification

#### 3.3.1 Baseline model

We employ a two-way fixed-effects model to analyze how improvements in household wealth perception capability influence elderly consumption. The benchmark model is specified as follows:


$$\ln(\text{consumptio}n_{\mathrm{ij}})=\beta_{0}+\beta_{1}\text{abilit}y_{\mathrm{ij}}+X_{\mathrm{ij}}+\gamma_{i}+\theta_{j}+\varepsilon_{\mathrm{ij}}$$ (2)

$$\ln(\text{consumptio}n_{\mathrm{ij}})=\beta_{2}+\beta_{3}\text{divid}e_{\mathrm{ij}}+X_{\mathrm{ij}}+\gamma_{i}+\theta_{j}+\varepsilon_{\mathrm{ij}}$$ (3)

In Equations 2, 3, consumption_ij_ denotes per capita consumption expenditure for the jth elderly household in year i; ability_ij_ denotes the wealth perception capability index of household j in year i, ${\text{divide}}_{\mathrm{ij}}$ denotes the wealth perception gap index of household j in year i. $X_{\mathrm{ij}}$ denotes the control variables, including household characteristics such as per capita net income, household head characteristics such as years of education, and regional characteristics such as regional per capita GDP. $\gamma_{i}$ denotes the year fixed effect. $\theta_{j}$ denotes the household fixed effect. $\varepsilon_{\mathrm{ij}}$ denotes the residual term.

#### 3.3.2 Mechanism analysis

Building on the classic approach to mediation-path identification developed by Baron and Kenny (1986) and further discussed by Agler and De Boeck (2017), this study employs a two-step strategy to examine two proposed mechanisms: perceived financial control and the reduction of excessive precautionary saving motives. The logic of this approach is that, for a variable to serve as a plausible mechanism linking the explanatory variable to the outcome, it should not only have a clear and theoretically meaningful connection to the outcome, but also be systematically associated with changes in the core explanatory variable. Put differently, a proposed mechanism can be regarded as empirically relevant only if the explanatory variable significantly predicts that mechanism. Accordingly, in Section 5, we first clarify, at the theoretical level, the psychological and behavioral pathways linking the two mechanism variables to the outcome variable. We then report OLS regression results for the association between the core explanatory variable and each mechanism variable in order to assess whether wealth perception capability is significantly related to these proposed psychological channels. The OLS model used in the mechanism analysis is specified as follows:

We first use the type of digital financial service used as the measure of perceived financial control and estimate an OLS model to examine this proposed channel. The specific model specification is as follows:


$$\begin{array}{l} \text{Type of Digital Financial}\;{\text{Service Used}}_{2021i}=\alpha_{0}\\ +\alpha_{1}\text{abilit}y_{2021i}+X_{2021i}+\varepsilon_{i} \end{array}$$ (4)

$$\begin{array}{l} \text{Type of Digital Financial}\;{\text{Service Used}}_{2021i}=\alpha_{2}\\ +\alpha_{3}\text{divid}e_{2021i}+X_{2021i}+\varepsilon_{i} \end{array}$$ (5)

In Equations 4, 5, Type of Digital Financial Service Used_2021i_ denotes the type of digital financial service used by the elderly household i in year 2021. The remaining variables are defined consistently with those in Equations 2, 3.

Second, we use digital financial payment preference as the measure of the reduction of excessive precautionary saving motives and likewise estimate an OLS model to test this proposed channel. The specific model specification is as follows:


$$\begin{array}{l} \text{Digital Financial}\;{\text{Payment Preference}}_{2021i}=\delta_{0}\\ +\delta_{1}\text{abilit}y_{2021i}+X_{2021i}+\varepsilon_{i} \end{array}$$ (6)

$$\begin{array}{l} \text{Digital Financial}\;{\text{Payment Preference}}_{2021i}=\delta_{2}\\ +\delta_{3}\text{divid}e_{2021i}+X_{2021i}+\varepsilon_{i} \end{array}$$ (7)

In Equations 6, 7, Digital Financial Payment Preferences_2021i_ denotes the digital financial payment preference of elderly household i in year 2021. The remaining variables are defined consistently with those in Equations 2, 3.

## 4 Empirical results

### 4.1 Baseline regression results

Table 3 presents the baseline regression results. Standard errors are adjusted for robust clustering, and all specifications include household fixed effects and year fixed effects. Column (1) reports the estimated effect of the household wealth perception capability index on elderly household consumption, while Column (2) reports the corresponding results using the household wealth perception gap index. The results show that the coefficient on the wealth perception capability index is positive and statistically significant at the 1% level, whereas the coefficient on the wealth perception gap index is negative and statistically significant at the 1% level. These findings indicate that, from both the absolute and relative perspectives of wealth perception, improvements in household wealth perception capability are significantly associated with an expansion of elderly household consumption, thereby providing empirical support for Hypothesis 1.

**Table 3:** Benchmark regression.

Variables	(1)	(2)
	Elderly Household Consumption	
Household Wealth Perception Capability Index	3.090***	
	(0.368)	
Household Wealth Perception Gap Index		−0.494***
		(0.061)
Household Income	0.037***	0.037***
	(0.006)	(0.006)
Household Assets	0.047***	0.047***
	(0.006)	(0.006)
Household Debt	0.005*	0.005*
	(0.003)	(0.003)
Household Size	−0.109***	−0.108***
	(0.010)	(0.010)
Age	−0.004	−0.004
	(0.005)	(0.005)
Health Status	0.025***	0.025***
	(0.009)	(0.009)
Marital Status	−0.050	−0.049
	(0.067)	(0.067)
Work Status	0.038*	0.034
	(0.022)	(0.022)
Gender	0.014	0.015
	(0.026)	(0.026)
Education Status	0.011***	0.010**
	(0.004)	(0.004)
Provincial Elderly Dependency	0.504	0.352
	(0.627)	(0.627)
GDP	−0.337	−0.247
	(0.306)	(0.306)
Provincial Tertiary Industry Value Added	0.366*	0.258
	(0.216)	(0.216)
Constant	8.992***	9.557***
	(2.351)	(2.354)
Control Variables	YES	YES
Year Fixed	YES	YES
Household Fixed	YES	YES
Observations	31,662	31,662
Adjusted *R*^2^	0.626	0.626

This table presents the benchmark regression results, where the explanatory variable in Column (1) is the Household Wealth Perception Capability Index, and the explanatory variable in Column (2) is the Wealth Perception Gap Index. Figures in brackets denote robust standard errors for household-level clustering; *, **, *** denote significance at the 10, 5, and 1% levels, respectively (* *p* < 0.1, ** *p* < 0.05, *** *p* < 0.01).

### 4.2 Endogeneity test

Elderly consumption is likely to be influenced by unobservable factors such as consumption preferences and attitudes. Moreover, improvements in household consumption levels may, in turn, enhance elderly households’ wealth perception capability, giving rise to potential reverse causality and biased estimates. To address these endogeneity concerns, this study employs an instrumental variable approach and a placebo test.

#### 4.2.1 Instrumental variable

This paper uses the regional volume of postal and telecommunications services at the end of 1984 as an instrumental variable for elderly households’ wealth perception capability. On the one hand, historical development in postal and telecommunications services is closely related to the current level of regional digital infrastructure and the wealth perception environment, thereby affecting households’ access to and use of wealth perception tools. On the other hand, as a form of communication infrastructure, historical postal and telecommunications activity has no direct bearing on the current consumption expenditure of elderly households, satisfying the exclusion restriction.

To address the potential mismatch in aggregation levels between historical macro-level variables and micro-level household data, following the related literature, we construct an interaction-based instrument by interacting historical postal and telecommunications volume with the average wealth perception capability index of other elderly households of the same age group, in the same community and survey year. Table 4 reports the two-stage least squares (2SLS) estimation results. The first-stage regressions show that the *F*-statistics for the wealth perception capability index and the wealth perception gap index are 736.021 and 451.961, respectively, indicating a strong correlation between the instrument and the endogenous regressors and effectively ruling out weak instrument concerns. The Kleibergen–Paap rk Wald LM statistics are significant at the 1% level, and the Kleibergen–Paap rk Wald F statistics exceed the conventional critical values at the 10% level, confirming that the model passes the under-identification and weak instrument tests and supporting the validity of the proposed instrument.

**Table 4:** Endogeneity test.

Variables	(1)	(2)	(3)
	Household Wealth Perception Capability Index	Elderly Household Consumption	
Total postal and telecommunications services volume at end-1984 × average wealth perception capability index of other households in the same community and age group during the same year	0.047***		
	(0.002)		
Household Wealth Perception Capability Index		21.096***	
		(1.049)	
Household Wealth Perception Gap Index			−4.639***
			(0.259)
Control Variables	YES	YES	YES
Year Fixed	YES	YES	YES
Household Fixed	YES	YES	YES
Observations	31,662	31,662	31,662
*F*-statistic	36.021	1183.492	894.854
KP rk LM statistic		666.861***	442.427***
KP rk Wald *F*-statistic		736.021	451.961

This table presents the results of the two-stage least squares (2SLS) regression using the regional volume of postal and telecommunications services at the end of 1984 as the instrumental variable. Column (1) shows the first-stage regression results of the instrumental variable on household wealth perception capability; Column (2) reports the second-stage regression results of the household wealth perception capability index on elderly household consumption; Column (3) reports the second-stage regression results of the household wealth perception gap index on elderly household consumption. Figures in brackets denote robust standard errors for household-level clustering; *, **, *** denote significance at the 10, 5, and 1% levels, respectively (* *p* < 0.1, ** *p* < 0.05, *** *p* < 0.01).

The second-stage results indicate that, after accounting for potential endogeneity, the household wealth perception capability index continues to exert a significantly positive effect on elderly households’ per capita consumption expenditure, while the household wealth perception gap index has a significantly negative effect. These findings are consistent with the baseline results.

#### 4.2.2 Placebo test

**Figure 1:**
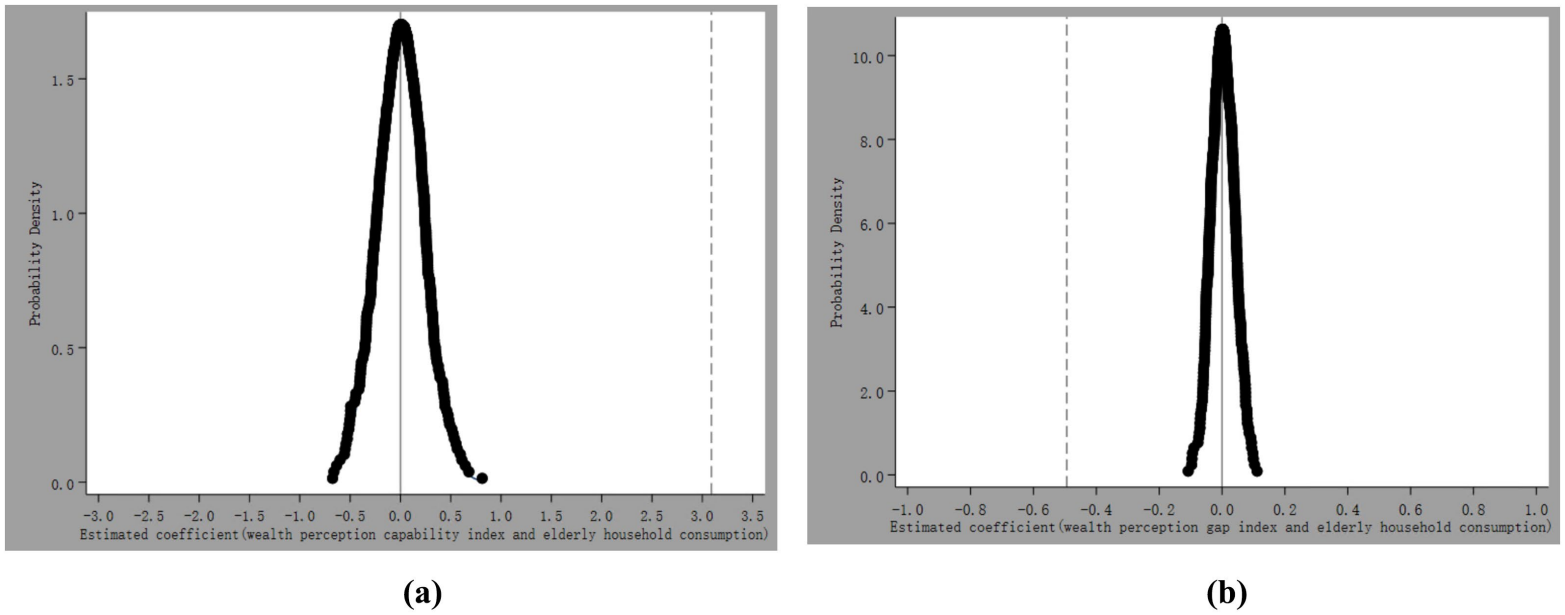
Distribution of estimated coefficients for the Household Wealth Perception Capability Index and Household Wealth Perception Gap Index based on 500 random samplings.

To further assess the robustness of the empirical findings, we conduct a placebo test by randomly generating the wealth perception capability index and the wealth perception gap index and repeating the baseline regressions 500 times. Figures 1a,b illustrate the distribution of the simulated coefficients. The results show that the mean of the simulated coefficients is close to zero and follows an approximately normal distribution, whereas the estimated coefficients based on the actual data lie well outside the simulated distributions. This evidence suggests that the baseline results are unlikely to be driven by random factors.

### 4.3 Robustness test

#### 4.3.1 Sample replacement

First, we exclude observations from first-tier cities. Major cities such as Beijing, Shanghai, Guangzhou, Shenzhen, and Hangzhou exhibit pronounced particularities in terms of wealth perception infrastructure, initial development conditions, and investment intensity. As a result, elderly households in these cities tend to possess substantially higher levels of wealth perception capability than those in other regions, which may unduly influence the estimated results. We therefore re-estimate the baseline regressions after excluding elderly households residing in these cities. The corresponding results are reported in Columns (1) and (2) of Table 5.

**Table 5:** Robustness checks: alternative samples.

Variables	(1)	(2)	(3)	(4)
	Elderly household consumption			
	Excluding first-tier cities		Balanced panel sample	
Household Wealth Perception Capability Index	3.821***		2.781***	
	(0.454)		(0.640)	
Household Wealth Perception Gap Index		−0.591***		−0.442***
		(0.073)		(0.102)
Constant	9.118***	9.892***	10.525**	11.170**
	(2.322)	(2.322)	(5.296)	(5.316)
Control Variables	YES	YES	YES	YES
Year Fixed	YES	YES	YES	YES
Household Fixed	YES	YES	YES	YES
Observations	27,352	27,352	3,429	3,429
Adjusted *R*^2^	0.593	0.592	0.563	0.563

This table presents the benchmark regression results after sample adjustment. Columns (1) and (2) use the sample excluding first-tier cities for regression. Columns (3) and (4) adopt the balanced panel sample for regression. Figures in brackets denote robust standard errors for household-level clustering; *, **, *** denote significance at the 10, 5, and 1% levels, respectively (* *p* < 0.1, ** *p* < 0.05, *** *p* < 0.01).

Second, we employ a balanced panel sample. While the baseline analysis is conducted using an unbalanced panel—which increases sample size and statistical power—missing observations for some households across survey waves may affect the stability of the fixed-effects estimates. To address this concern, we further re-estimate the model using a balanced panel consisting of households observed in all three survey waves from 2017 to 2021. The results are presented in Columns (3) and (4) of Table 5. In both cases, improvements in household wealth perception capability continue to significantly promote elderly household consumption, indicating that the baseline findings remain robust to alternative sample constructions.

#### 4.3.2 Alternative variable measurement

First, we adjust indicator weights. The wealth perception capability index constructed in the baseline analysis is applicable to the full sample. However, older adults may exhibit distinct patterns in technology use and participation in wealth perception activities. Accordingly, we recalculate the weights of the underlying indicators using only the elderly household subsample and reconstruct both the wealth perception capability index and the wealth perception gap index. The baseline regressions are then re-estimated, with results reported in Columns (1) and (2) of Table 6.

**Table 6:** Robustness checks: alternative measurement methods.

Variables	(1)	(2)	(3)	(4)
	Elderly Household Consumption			
Household Wealth Perception Capability Index (reweighted entropy)	2.452***			
	(0.260)			
Household Wealth Perception Gap Index (reweighted entropy)		−0.527***		
		(0.056)		
Household Wealth Perception Capability Index (calculated using factor analysis)			0.579***	
			(0.058)	
Household Wealth Perception Gap Index (calculated using factor analysis)				−0.221***
				(0.047)
Constant	8.941***	9.530***	8.533***	9.486***
	(2.348)	(2.347)	(2.351)	(2.356)
Control Variables	YES	YES	YES	YES
Year Fixed	YES	YES	YES	YES
Household Fixed	YES	YES	YES	YES
Observations	31,662	31,662	31,662	31,662
Adjusted *R*^2^	0.627	0.627	0.628	0.624

This table presents the regression results after adjusting the measurement method of the Household Wealth Perception Capability Index. The explanatory variables in Columns (1) and (2) are the Household Wealth Perception Capability Index with weights of each indicator recomputed based on the elderly household sample. The explanatory variables in Columns (3) and (4) are the Household Wealth Perception Capability Index calculated by the factor analysis method. Figures in brackets denote robust standard errors for household-level clustering. *, **, *** denote significance at the 10, 5, and 1% levels, respectively (* *p* < 0.1, ** *p* < 0.05, *** *p* < 0.01).

Second, we adopt an alternative measurement method. As the entropy-weighting method does not explicitly account for the correlation structure among indicators, we further employ factor analysis to construct the household wealth perception capability index and the wealth perception gap index, and re-estimate the model accordingly. The results are shown in Columns (3) and (4) of Table 6. Across different weighting schemes and measurement approaches, improvements in household wealth perception capability consistently lead to a significant expansion in elderly household consumption, thereby confirming the robustness of the results.

#### 4.3.3 Variable substitutions

First, we replace the dependent variable. Although medical expenditures are relatively rigid, they may still be influenced by wealth perception capability. We therefore use per capita household consumption, including medical expenditures, as an alternative dependent variable. The corresponding results are reported in Columns (1) and (2) of Table 7.

**Table 7:** Additional robustness checks.

Variables	(1)	(2)	(3)	(4)	(5)	(6)
	Elderly Household Consumption					
	Alternative Dependent Variable		Controlling for City Fixed Effects		Adjusting Cluster Standard Errors	
Household Wealth Perception Capability Index	2.787***		3.094***		3.090***	
	(0.343)		(0.369)		(0.728)	
Household Wealth Perception Gap Index		−0.402***		−0.495***		−0.494***
		(0.057)		(0.061)		(0.121)
Constant	10.559***	11.032***	8.931***	9.503***	8.992*	9.557*
	(2.202)	(2.205)	(2.362)	(2.364)	(5.249)	(5.216)
Control Variables	YES	YES	YES	YES	YES	YES
Year Fixed	YES	YES	YES	YES	YES	YES
Household Fixed	YES	YES	YES	YES	YES	YES
Observations	31,662	31,662	31,662	31,662	31,662	31,662
Adjusted *R*^2^	0.625	0.624	0.617	0.617	0.626	0.626

This table presents the results of other robustness tests. The dependent variables in Columns (1) and (2) are per capita consumption expenditure including medical expenditures. Columns (3) and (4) control for city fixed effects. Columns (5) and (6) adjust the standard errors to the community level with clustering. Figures in brackets denote robust standard errors for household-level clustering. *, **, *** denote significance at the 10, 5, and 1% levels, respectively (* *p* < 0.1, ** *p* < 0.05, *** *p* < 0.01).

Second, we control for city fixed effects. Given that elderly household consumption may be affected by regional differences in pension policies and social security systems, we further include city fixed effects in the regression. The results are presented in Columns (3) and (4) of Table 7.

Third, we adjust the clustering level of the standard errors. To mitigate potential bias arising from unobserved correlations at the community level, standard errors are clustered at the community level. The corresponding estimates are reported in Columns (5) and (6) of Table 7.

Overall, after replacing variables, modifying fixed-effects specifications, and adjusting the clustering dimension, the positive effect of household wealth perception capability on elderly consumption remains statistically significant, providing further support for the robustness of the baseline regression results.

## 5 Mechanism analysis

According to life-cycle theory, the accumulation of household wealth generally helps promote consumption decisions. However, household consumption decisions are not determined solely by objective wealth levels; individuals’ subjective judgments regarding the availability and liquidity of wealth, as well as future financial security, also play a critical role (Kivetz, 1999). For older adults, such subjective psychological evaluations are especially important. On the one hand, as individuals move into the later stages of the life cycle, they face higher longevity risk and greater uncertainty, making their consumption decisions more susceptible to precautionary motives. On the other hand, with increasing age, individuals often face greater constraints in information processing, financial cognition, and risk assessment, and therefore rely more heavily on subjective perceptions of their resource conditions when making consumption decisions. From this perspective, perceived financial control reflects individuals’ subjective sense of whether their financial resources are controllable and disposable, whereas the reduction of excessive precautionary saving motives captures the extent to which individuals refrain from suppressing current consumption because of excessive concern about future uncertainty. Together, these two factors constitute important subjective psychological channels through which consumption decisions among older adults are shaped.

We first use the type of digital financial service household used to measure perceived financial control. The rationale is that, compared with traditional payment methods such as cash and bank cards, digital financial platforms typically integrate multiple functions, including saving, payment, and investment, allowing individuals to grasp their overall financial situation through a more intuitive interface. For older adults, a greater variety of accessible and usable digital financial accounts implies more channels through which wealth-related information can be obtained and clearer perceptions of the availability and disposability of assets, thereby making it easier to develop a stronger sense of financial control. Accordingly, the variety of digital financial accounts serves as a useful proxy for differences in perceived financial control among older adults. Specifically, if a household’s commonly used payment methods include one, two, or all three of WeChat Pay, Alipay, and mobile banking, the variable is coded as 1, 2, or 3, respectively.

Second, we use households’ digital financial payment preference to measure the reduction of excessive precautionary saving motives. To some extent, digital payment preference reflects older adults’ subjective expectations regarding future payment convenience, access to funds, and the security of everyday transactions. For older adults, a stronger preference for using mobile payment in place of bank cards and cash suggests a higher level of trust in and adaptation to digital financial tools. It also implies weaker psychological barriers to the use of wealth in daily life and lower perceived uncertainty regarding future transactions and payments. Correspondingly, greater acceptance of digital payment is likely to weaken excessive precautionary saving motives driven by uncertainty, thereby releasing current consumption potential. Digital payment preference can therefore, to some extent, reflect reducing excessive precautionary saving motives. Specifically, if the respondent agrees that a mobile phone can substitute for bank cards and wallets, the variable is coded as 1; otherwise, it is coded as 0.

Because these two mechanism variables are available only in the 2021 wave of the China Household Finance Survey (CHFS), the empirical analysis in this subsection is based on cross-sectional data from 2021. The results reported in Table 8 show that the effects of household wealth perception capability on both the variety of digital financial accounts and digital payment preference are positive and statistically significant at the 1% level. These findings indicate that improvements in household wealth perception capability significantly strengthen older adults’ perceived financial control and reduce their excessive precautionary saving motives, thereby contributing to an expansion in elderly household consumption.

**Table 8:** Mechanism analysis.

Variables	(1)	(2)	(3)	(4)
	Type of Digital Financial Service Used		Digital Financial Payment Preference	
Household Wealth Perception Capability Index	10.012***		1.096***	
	(0.788)		(0.397)	
Household Wealth Perception Gap Index		−1.199***		−0.131***
		(0.094)		(0.048)
Constant	−1.469***	−0.265	0.946***	1.078***
	(0.442)	(0.461)	(0.222)	(0.231)
Control Variables	YES	YES	YES	YES
Observations	3,695	3,695	3,695	3,695
Adjusted *R*^2^	0.191	0.191	0.057	0.057

This table presents the regression results of Household Wealth Perception Capability Index on the type of digital financial service used and digital financial payment preference of elderly households. The dependent variable in Columns (1) and (2) is the type of digital financial service used by elderly households; the dependent variables in Columns (3) and (4) are digital payment preference. Figures in brackets denote robust standard errors for household-level clustering. *, **, *** denote significance at the 10, 5, and 1% levels, respectively (* *p* < 0.1, ** *p* < 0.05, *** *p* < 0.01).

## 6 Heterogeneity analysis

### 6.1 Family structure

Based on household composition, the sample is divided into pure elderly households and mixed elderly households. Pure elderly households consist exclusively of older members whereas mixed elderly households are headed by an older adult and co-reside with younger family members. Compared with pure elderly households, elderly household heads in mixed households are more likely to be influenced by the consumption attitudes and behaviors of younger members, and thus tend to exhibit greater adaptability and diversity in consumption choices and consumption modes. Under these circumstances, improvements in household wealth perception capability are more likely to affect consumption decisions in mixed elderly households through intergenerational interaction and information transmission within the family. Accordingly, we introduce a dummy variable, mixed, which equals 1 if the elderly household is a mixed household and 0 if it is a pure elderly household. Column (1) of Table 9 reports the regression results for the interaction between household wealth perception capability and the mixed-household dummy. The interaction term is positive and statistically significant at the 1% level, indicating that the consumption-promoting effect of wealth perception capability is stronger for mixed elderly households.

**Table 9:** Heterogeneity analysis.

Variables	(1)	(2)	(3)
	Elderly Household Consumption		
Household Wealth Perception Capability Index	1.864***	2.220***	4.502***
	(0.455)	(0.391)	(0.821)
Household Wealth Perception Capability Index × Non-Pure Elderly Households	2.289***		
	(0.601)		
Household Wealth Perception Capability Index × Rural Areas		4.164***	
		(0.917)	
Household Wealth Perception Capability Index × High Self-Perceived Health Level			−1.693*
			(0.874)
Constant	8.993***	9.103***	8.902***
	(2.352)	(2.347)	(2.349)
Control Variables	YES	YES	YES
Year Fixed	YES	YES	YES
Household Fixed	YES	YES	YES
Observations	31,662	31,662	31,662
Adjusted *R*^2^	0.627	0.627	0.626

This table presents the heterogeneous regression results of Household Wealth Perception Capability on elderly household consumption. The explanatory variable in Column (1) is the interaction term between Household Wealth Perception Capability Index and mixed elderly households. The explanatory variable in Column (2) is the interaction term between Household Wealth Perception Capability Index and rural areas. The explanatory variable in Column (3) is the interaction term between Household Wealth Perception Capability Index and household heads with high self-perceived health. *, **, *** denote significance at the 10, 5, and 1% levels, respectively (* *p* < 0.1, ** *p* < 0.05, *** *p* < 0.01).

### 6.2 Urban and rural

We introduce a dummy variable, rural, which equals 1 if an elderly household resides in a rural area and 0 otherwise. Column (2) of Table 9 reports the regression results for the interaction between household wealth perception capability and the rural dummy. The coefficient on the interaction term is positive and statistically significant at the 1% level, indicating that, relative to urban areas, improvements in wealth perception capability exert a more pronounced consumption-enhancing effect on rural elderly households.

### 6.3 Self-perceived health level

Self-rated health influences individuals’ subjective expectations regarding future survival, which in turn affects saving and consumption decisions. In general, older adults with better self-rated health tend to have higher expected survival probabilities and, consequently, stronger precautionary saving motives. This may attenuate the marginal effect of improvements in wealth perception capability on their consumption behavior. To test this mechanism, we introduce a dummy variable capturing the household head’s self-rated health status. Households in which the household head reports his or her health status as “fair,” “good,” or “very good” are classified as having high self-rated health and assigned a value of 1, while the remaining households are classified as having low self-rated health and assigned a value of 0. Column (3) of Table 9 reports the regression results for the interaction between household wealth perception capability and the high self-rated health dummy. The coefficient on the interaction term is negative and statistically significant at the 10% level, indicating that, relative to elderly households with higher self-rated health, improvements in wealth perception capability have a more pronounced consumption-enhancing effect among elderly households with lower self-rated health.

## 7 Conclusion

Adopting a micro-level perspective centered on household wealth perception capability, this study empirically examines its impact on decision-making among elderly households using data from the 2017, 2019, and 2021 waves of the China Household Finance Survey (CHFS). The main findings are as follows. First, improvements in wealth perception capability significantly enhance the proactivity of consumption decision-making among elderly households. This result remains robust after addressing endogeneity concerns through an instrumental variable approach, placebo tests, and a series of robustness checks, including sample adjustments, alternative measurements of key variables, variable substitutions, and clustered standard-error corrections. Second, mechanism analysis indicates that wealth perception capability promotes more active decision-making primarily by strengthening perceived financial control and reducing excessive precautionary saving motives among older adults. Third, heterogeneity analysis further reveals that the decision-enhancing effect of wealth perception capability is more pronounced among elderly households with higher levels of psychological vulnerability.

Our findings also have important practical implications. Against the backdrop of deepening population aging and rapid digital development, efforts to promote consumption among older adults should not rely solely on objective conditions such as income growth or increases in wealth, but should also pay greater attention to older adults’ ability to perceive, understand, and use their existing financial resources. More specifically, further efforts are needed to advance age-friendly transformation, simplify the presentation of information, help older adults strengthen their sense of control over their financial resources, and alleviate excessive psychological caution arising from uncertainty about the future. More broadly, improving wealth perception capability may not only enhance consumption participation among older adults, but also strengthen their sense of financial security and decision-making autonomy in later life.

This study also has several limitations. First, the core mechanism variables are available only in the 2021 wave of the CHFS, and the mechanism analysis is therefore based primarily on cross-sectional data, which does not allow the dynamic evolution of the relevant psychological mechanisms to be fully identified. Second, although we identify several important sources of heterogeneity, older adults still differ in more complex ways in terms of vulnerability and neurological conditions, which deserve further investigation. Future research may draw on panel data covering a longer time span or follow-up survey data to examine more rigorously the dynamic relationship between wealth perception capability and consumption decision-making among older adults. In addition, future studies may further explore whether wealth perception capability influences other important outcomes in later life, such as mental health and changes in neurological functioning.

## Data Availability

The datasets presented in this article are not readily available because the authors are not permitted to share data. Requests to access the datasets should be directed to https://chfs.swufe.edu.cn/.
